# Divide and Conquer: Russia's Anti-west Campaign and Gender-based Disinformation

**DOI:** 10.1177/00207020261452284

**Published:** 2026-05-25

**Authors:** Owen Wong, Claire Mountford, Stéfanie von Hlatky

**Affiliations:** Queen's University, Kingston, Ontario, Canada; 4257 Queen's University, Kingston, Ontario, Canada; 4257 Queen's University, Kingston, Ontario, Canada

**Keywords:** influence operations, NATO, Russia gender-based disinformation, disinformation

## Abstract

This article investigates how Russia employs gender-based disinformation as a strategic tool to advance its foreign policy objectives. Although existing research on gender-based foreign influence and disinformation emphasizes its domestic social effects, it has not fully explored how states instrumentalize gendered narratives to pursue broader geopolitical goals. Using qualitative content analysis of 367 pro-Kremlin disinformation articles, we identify eight narratives that Russia employs to undermine the moral legitimacy of Western states and institutions, such as NATO and the EU, while simultaneously legitimizing its own actions. We show that Russia strategically deploys disinformation to resonate with conservative audiences. Our analysis highlights two core mechanisms: legitimacy sabotage and legitimation, both of which are rooted in the contestation of moral authority. By weaponizing gendered narratives, Russia effectively reconfigures legitimacy landscapes in its favour, illustrating the broader strategic logic of gender-based disinformation within contemporary hybrid warfare tactics. This research presents a novel framework for understanding how states use identity-based narratives in the context of great power competition.

Scholars and defence practitioners have become interested in disinformation's role in contemporary great power competition.^1^ In December 2025, NATO's Secretary General Mark Rutte warned that Russia could attack a NATO member-state within the next five years.^2^ In his discussion with Germany's Foreign Affairs Minister, he stated, “Russia is already escalating its covert campaign against our societies.”^3^ Despite evidence that states use gender-based disinformation to shape domestic politics and reinforce social biases, less attention has been given to how states weaponize these gendered narratives beyond their borders.

Throughout this article, we ask: How does Russia use gender-based disinformation as a hybrid tactic to achieve its foreign policy objectives? We suggest that gender-based disinformation is not just a tool that has domestic social impacts. Instead, Russia uses gender-based disinformation to advance its geopolitical objectives by undermining its adversaries’ moral legitimacy and strengthening the legitimacy of its own goals. Specifically, we argue that Russian gender-based disinformation serves three primary goals: undermining the cultural influence of Russia's adversaries, eroding public support for their political institutions, and legitimizing Russia's foreign policy actions.

Although states, including China and Iran, use gender-based disinformation to discredit public dissidents, the extant literature suggests that Russia is the largest and most serious offender.^4,5^ Accordingly, we draw on a dataset of 367 pro-Kremlin disinformation articles to identify the main narratives and mechanisms they rely on. We show that Russia uses gender-based disinformation to undermine the cultural influence of Western liberal democracies, contain NATO and the EU, and justify its military aggression to conservative audiences in Central and Eastern Europe (CEE). Our analysis highlights the strategic logic of gender-based disinformation within contemporary great power competition, opening new avenues for research on gender, disinformation, and international security.

## Gender-based disinformation

Disinformation is false information that an actor spreads with the intent to deceive.^6^ It is a subset of misinformation, which is false information that is spread by an actor with or without the intent to deceive. The primary distinction between disinformation and misinformation is the actor's intent. Although proving that an actor has the intent to deceive its audience is difficult, one may assume intent if there is evidence of organized attempts by a political actor to spread misinformation.^7^

Gender-based disinformation is lower on the ladder of abstraction, meaning that it is a specific subtype of disinformation. It is characterized by false narratives that evoke traditional constructs of masculinity and femininity to serve the propagator's political, social, and economic goals.^8^ It may be a particularly powerful type of disinformation because it weaponizes pre-existing stereotypes about gender and sexuality to influence public opinion. Scholars studying gender and politics have written extensively about how gender-based disinformation influences women's political engagement^9^ and may be reflected in the political coverage of female politicians.^10^ Gender-based disinformation may also incite gender-based violence^11^ and normalize forms of sexual violence.^12^

Although the literature on gender-based disinformation explores its dynamics in domestic contexts, it has largely overlooked how states weaponize these narratives to achieve foreign policy objectives. We maintain that gender-based disinformation is not merely a tool of domestic political suppression but also a strategic instrument of statecraft, used to weaken adversaries’ legitimacy, sow discord, and project alternative value systems. By weaponizing gendered narratives, states subtly challenge liberal norms, the moral legitimacy of strategic competitors, and advance foreign policy agendas under the guise of cultural preservation or moral superiority.

Some scholars highlight how states use gender norms to advance their political goals but stop short of analyzing disinformation. For example, Laura Sjoberg highlights that states often justify military aggression by feminizing their adversaries and claiming that they need to be protected or treasured.^13^ States also spread misogynistic narratives about female candidates in other countries, which under special conditions, can help the candidate overcome structural disadvantages.^14^ In a study of over 7,000 tweets from state-sponsored accounts in Russia, Iran, and Venezuela, the authors found that foreign state actors co-opted intersectional critiques of feminism and female empowerment to polarize foreign publics and undermine support for female candidates.^15^ States also use gendered norms as a cognitive warfare tactic to deepen divisions among NATO members, undermine support for gender equality, and exacerbate social divisions. ^16^

Although feminizing adversaries, spreading misogynistic narratives, and engaging in cognitive warfare may help political actors advance their goals, to avoid conceptual stretching, we maintain that these are not necessarily examples of gender-based disinformation. For example, statements like “women shouldn’t be leaders” are misogynistic and normative expressions of prejudice. However, they are conceptually different from disinformation, which necessarily contains false claims about facts. For example, a statement claiming that “women shouldn’t be leaders because they are not as intelligent as men” is a form of gender-based disinformation. Furthermore, evidence that Russia co-opts critiques of the feminist movement to fragment solidarity among feminists does not meet the requirements of gender-based disinformation. For example, Bradshaw and Henle argue that Russian bots and trolls spread narratives that the feminist movement is too white to represent Black feminists.^17^ Although Russia deliberately amplifies these critiques, they are not necessarily false and therefore cannot be considered disinformation. Indeed, Bradshaw and Henle carefully use the term “foreign influence campaign” to capture this nuance.^18^

This study focuses squarely on state-aligned gender-based disinformation. The “state-aligned” modifier on disinformation is included to describe both disinformation created by a state and disinformation created by actors who use disinformation to promote the state's foreign policy objectives.^19^ Although disinformation, weaponized misogyny, and influence campaigns may all be harmful, disinformation is particularly pernicious because it seeks to persuade its audience through claims that are presented as factual but are untrue or misleading. While all states use different techniques to influence foreign citizens, such as public diplomacy or strategic communications, disinformation is uniquely malicious because it corrupts the information environment and often distorts grains of the truth to misrepresent an actor in ways that seem credible or authoritative.

We conceptualize state-sponsored gender-based disinformation as a hybrid tactic. Hybrid tactics deliberately blur the lines between peace and conflict, combining conventional and unconventional means (such as cyber tools, psychological operations, and disinformation) to achieve strategic objectives without triggering a direct military response. These concerns have been recognized by the chief of MI6, Blaise Metreweli, who noted that Russia is “testing us in the grey zone with tactics that are just below the threshold of war.”^20^ According to Metreweli, “The export of chaos is a feature, not a bug, in this Russian approach to international engagement.”^21^ By framing gender-based disinformation as a hybrid tactic, we highlight how it functions not merely as a tool for domestic manipulation, but as part of a broader strategy to weaken adversaries’ cohesion, undermine democratic legitimacy, and project alternative normative orders. This perspective highlights that gender-based disinformation is connected to geopolitical competition and is not just a domestic social issue.

Concerns with state-sponsored and affiliated gender-based disinformation are also prominent in policy circles. NATO's 2024 Women, Peace, and Security (WPS) policy highlights how strategic competitors use gendered disinformation to undermine the social cohesion of democracies and exacerbate polarization.^22^ At the 2024 Open Debate on WPS at the United Nations, thirty percent of countries in attendance highlighted the security threats posed by either technology or disinformation in their three-minute statements.^23^ Despite growing recognition from actors like NATO and Canada that technology-facilitated gender-based violence and gendered disinformation are important security threats, the academic literature lacks an adequate assessment of the way states use gender-based disinformation to promote their foreign policy goals. This research addresses that gap by theorizing gender-based disinformation not merely as a domestic phenomenon, but as a strategic instrument of statecraft with international security implications.

## Gender-based disinformation as a hybrid tactic

Gender-based disinformation is a hybrid tactic that states like Russia, China, and Iran use to enhance their capacity and constrain their adversaries without crossing the threshold of conflict.^24^ In the domestic context, scholars have written about how women in politics and feminist movements are targets of gender-based disinformation.^25^ By contrast, we focus on states like Russia that deliberately disseminate false information that weaponizes norms surrounding gender, sexuality, and sexual violence as part of a coherent strategy to constrain foreign adversaries and expand their influence.

We distinguish state-affiliated gender-based disinformation, which relies on intentional falsehoods, from influence campaigns that seek to foment unrest by strategically amplifying truthful claims about gender and sexuality. For example, the Government of Canada publicly amplified Iranian women's voices during the 2022 protests against Iran's violent enforcement of mandatory veiling laws.^26^ Canada's criticism of the Iranian government was grounded in verifiable facts and therefore does not constitute gender-based disinformation. This stands in sharp contrast to claims circulated by pro-Kremlin outlets – such as assertions that the EU is “imposing gender neutrality on Georgian kindergartners.”^27^ Such statements constitute gender-based disinformation because they are demonstrably false, disseminated by proxy actors with the intent to deceive, and designed to exploit anxieties surrounding gender and sexuality.

Gender-based disinformation manipulates ideas at the intersection of gender and identity. Gender roles are pervasive, ingrained norms that dictate how biology should influence the way people behave. These roles are a product and tool of socialization and inform the shared expectations of how people of a certain gender should act, including their social positions or categories.^28^ Although gender can be malleable, norms about what is socially acceptable or not often influence the way people express these identities. Gender roles impact both individual identities and the way individuals act within society. Gender roles manifest culturally in terms of “situational constraints” that effectively normalize behaviour associated with one gender or another.^29^ In the case of gendered disinformation, both traditional gender roles and norms work with disinformation to uphold narratives that advance specific foreign policy goals.

Gender-based disinformation may be particularly pernicious because it taps into its audiences’ social identities.^30^ Research on motivated reasoning consistently shows that people are more likely to believe information if it aligns with their social identity or moral values. For example, researchers found that people who were morally opposed to education about contraceptives were more likely to believe that condoms were ineffective at preventing sexually transmitted infections.^31^ Similarly, people who thought capital punishment was immoral were less likely to believe that it was effective at preventing crime.^32^ As these examples illustrate, moral commitments influence how individuals process information, biasing belief formation toward identity-consistent conclusions rather than accuracy. Gender-based disinformation leverages identity-consistent moral reasoning to make disinformation appear plausible, even in the absence of supporting evidence.

States like Russia use gender-based disinformation to strategically influence public opinion. These states seek to make their goals appear more legitimate to domestic and foreign audiences. They also seek to make their adversaries appear less legitimate to domestic and foreign audiences. According to Schuman, legitimacy is the “perception or assumption that the actions of an entity are desirable, proper, or appropriate within some socially constructed systems of norms, values, beliefs, and definitions.”^33^ When a state's actions are legitimate, they are perceived as acceptable and justified “in the eyes of other states and their citizens.”^34^

Legitimacy is an important resource in international relations because it shapes the willingness of citizens, other states, and foreign nationals to support, comply with, or resist a country or an international organization's policies. In 2004, the neoconservative Robert Kagan suggested that “the struggle to define and obtain international legitimacy in this new era may prove to be among the most critical contests of our time.”^35^ Kagan understood that legitimacy is intrinsically connected to power.^36^ In the language of material capabilities, legitimacy can make it easier for a state to acquire resources such as troop contributions and weapons, domestic compliance with foreign policy goals, and may lower the likelihood that a state will face sanctions and other forms of opposition for its actions.^37^ T.V. Paul summarizes these dynamics when he suggests that legitimacy lowers the cost of exerting power for states of all sizes.^38^ In the context of alliances, he maintains that sustaining an alliance and acquiring new members requires legitimacy. Although military power can help acquire new members, coalitions based on strength alone are unlikely to last. In short, when a foreign policy goal is widely perceived as legitimate, it becomes easier for a state or international organization to exercise its power. When a course of action is perceived as illegitimate, actors of all types must contend with greater constraints.

We focus on Russia, which uses gender-based disinformation to sabotage the legitimacy of its geopolitical adversaries in two ways. First, Russia uses gender-based disinformation to undermine the cultural influence of its adversaries. Russia strategically targets conservative audiences with gender-based disinformation, claiming that countries like the US are too progressive and therefore culturally incompatible with the values of conservative audiences. These narratives provide a compelling moral reason for conservatives exposed to disinformation to want to distance themselves from these culturally incompatible states.

Russia also seeks to undermine the legitimacy of international organizations. It spreads disinformation, claiming that the progressive gender values these organizations champion will lead to negative outcomes. For example, Russia regularly claims that if countries like Georgia develop closer ties with international organizations like NATO and the EU, it will lead to the erosion of traditional family values. Russia also accuses its adversaries of hypocrisy. It spreads false narratives that claim that its adversaries appear to champion gender equality but are, in fact, immoral. For example, Russia claims that NATO and the EU appear to champion gender equality but are in fact regular perpetrators of sexual violence. The goal of this type of disinformation is to foster institutional skepticism among citizens of NATO and EU member states, as well as future member states.

Russia also uses gender-based disinformation to make its own foreign policy goals appear more legitimate. These legitimation strategies appeal to commonly held conservative values. For example, Russia might suggest that its citizens and those in its rival's country should join forces to fight against foreign-imposed progressive values. When these narratives are directed toward citizens outside of Russia, they suggest that the two countries should work together to preserve traditional family values and defend against the “woke gender ideology” that is being professed by countries like Canada and the US. In some cases, this narrative may even be used to justify direct military intervention to protect citizens from foreign states intent on eroding traditional values. In short, gender-based disinformation can help cultivate like-mindedness in states with similar ideals, which in turn fosters stronger relationships by appealing to the idea of fighting together against progressive values.

Russia also uses gender-based disinformation to protect its legitimacy from accusations of war crimes or gross wrongdoings. Russia may spread disinformation to counter these accusations in two ways. First, it suggests that accusations of crimes like conflict-related sexual violence are fabricated. Second, it uses gender-based ad hominem attacks to undermine the character of individuals or institutions, accusing them of war crimes or gross wrongdoings. For example, these attacks might suggest that someone accusing a state of wrongdoing cannot be trusted because they themselves are perpetrators of sexual violence. This kind of ad hominem attack is a useful logical fallacy because it allows Russia to defend against accusations without providing evidence that the accusation is baseless.

Although we argue that gender-based disinformation can be an effective hybrid tactic, its success is not guaranteed. The effectiveness of these narratives depends on local norms, media ecosystems, and levels of societal polarization. In places where progressive norms are strong and audiences have strong media literacy skills, these narratives may be more easily rejected.

## Research design

Our research process consisted of three phases. The first phase involved gathering media articles from the “EU vs Disinfo” database, which is publicly available on the EU's website.^39^ The database aims to identify and itemize various pieces of media published by pro-Kremlin sources, which are then classified as disinformation using a “disproof”, or an explanation of which component of the article specifically contains misleading or false information. Throughout this article, we use the term “Russian disinformation” to refer to these articles. We surveyed the literature on gender-based disinformation to compile a list of keywords that would likely yield articles discussing gender-related issues. We tested each keyword individually, discarding keywords that produced either no results in our dataset or too many results that did not substantively discuss gender-related issues. We selected keywords that relate to three dimensions of gender-related issues: sexual violence, sexuality, and gender identity and expression. To capture articles about sexual violence, we used the keywords “rape”, “sexual violence” and “sexual assault”. To identify articles related to sexuality, we chose the keywords “gay” and “LGBT”. The keywords “masculine”, “feminine”, “gender”, and “family values” were selected for the gender identity and expression category. We excluded the keyword “trans” because “trans” is captured by the “T” in “LGBT,” and “trans” returned mostly articles about Transnistria.

After the corpus was assembled, both coders assembled a sample of twenty-five articles.^40^ Each article was coded into a narrative category until no new categories remained. Then, each coder took a second sample of ten articles and tested the coding categories. In total, sixty articles were used to develop the eight narrative categories. Coders manually checked to make sure each article included the relevant keyword. Articles that did not contain a relevant keyword, but were retrieved by the search engine (false positives), were coded by two coders and discarded if both coders agreed the article was a false positive. In most cases the search engine returned false positives when one of the keywords was contained in the “read similar articles” section of the webpage. Once all articles were coded, the dataset was cleaned in R to eliminate duplicate articles. In total, 520 articles were retrieved and coded from the EU vs Disinfo website based on the nine keywords derived from the literature. Because there was substantial overlap between the articles produced by each keyword, 153 duplicate articles were dropped. Beyond recording descriptive information about the articles, each coder determined the article's prevailing narrative based on eight categories, described in our empirical section.

The greatest drawback of this dataset is that it does not accurately reflect the frequency or trends in Russian gender-based disinformation across time. This is a limitation that stems from the nature of the EU vs Disinfo dataset; the dataset focuses on disproving disinformation from multiple sources, rather than monitoring and identifying each disinformation article from a stable set of sources. Given the limitations of this dataset, this article proposes to qualitatively analyze the content of gender-based disinformation to better understand contemporary hybrid tactics. Future research could focus on the development of an original dataset to test the frequency of the strategies and narratives discussed here.

## Russian gender-based disinformation

Beginning in 2007, Russia started using a series of covert hybrid warfare strategies to weaken Western states and institutions from within.^41^ In 2013, these tactics garnered public attention when General Gerasimov, Russia's Chief of the General Staff, stated that non-military methods were becoming an increasingly important means for Russia to achieve its military and strategic objectives.^42^ Russia then targeted the US, as well as institutions such as NATO and the EU, which are both staunch promoters of democracy and have the combined capability to limit Putin's revisionist geopolitical ambitions. After the colour revolutions in Georgia and Ukraine, Putin became worried that a democratic contagion could threaten his regime. Beyond the political threat that NATO and the EU pose to Russia, Putin became worried that further economic cooperation with the EU would threaten Russia's model of crony capitalism. For instance, Light argues that Russia's decade of economic cooperation with the EU ended in the mid-2000s because of economic and administrative incompatibilities.^43^ Rather than continuing cooperative engagement with the EU, Russia encouraged or put pressure on states to join its Eurasian Economic Union (EEU) to compete with the EU's supremacy.

Through its hybrid tactics against Western states, Russia is simultaneously pursuing three goals. First, it seeks to undermine, weaken, and eventually destroy NATO and the EU by fomenting anti-NATO sentiment and Euroscepticism. Weakening these international organizations would “halt their attempts to integrate former Soviet republics and satellite states and strengthen Russia's hand with its former imperial subjects in CEE.”^44^ Russia also seeks to increase its regional influence by becoming the primary supplier of oil and gas in Europe. Finally, it seeks to reduce the ties that the EU has with the US, a key NATO member and a historically strong advocate of democracy.

Russia also uses a series of covert hybrid tactics to undermine Western liberal democracies. It funds extremist Eurosceptic parties, finances politicians who are sympathetic to Russian foreign policy goals, and supports political campaigns that might draw voters away from mainstream candidates.^45^ For example, Russia may have provided funding to the Brexit “Leave” movement, and during Marine Le Pen's 2017 presidential bid, Russian banks with connections to the Kremlin helped to finance her campaign.^46^ Cyber-attacks are also a mainstay in Russia's hybrid warfare arsenal.^47^ In the 2004 Ukrainian Presidential election, Russia hacked into the Central Election Commission to change the election results, triggering a rerun election that brought a pro-Russian candidate into office.^48^

Most relevant to our analysis is the role of disinformation and propaganda. Russia regularly uses disinformation to divide civilians, undermine their trust in democratic processes and institutions, and erode their support for international organizations like NATO and the EU.^49^ These media campaigns often present distorted or selectively framed narratives that amplify existing social divisions, spread misinformation, and promote anti-Western sentiment. By leveraging culturally tailored messaging and exploiting existing grievances within target populations, Russia seeks to delegitimize liberal democratic values and weaken the cohesion of Western alliances, thereby advancing Russia's strategic interests without direct military confrontation.

As further detailed below, our empirical analysis suggests that Russia uses gender-based disinformation to undermine Western states and institutions in two ways. First, it uses gender-based disinformation to undermine the legitimacy of the ideology of Western liberal democracies and their institutions. Second, it uses gender-based disinformation to strengthen the legitimacy of its own foreign policy goals. Consistent with our proposed theoretical mechanism, Russia focuses on the moral legitimacy of its worldview and contrasts it with narratives about the West's moral corruption. By tapping into commonly held conservative values, Russia justifies its foreign policy goals and portrays the values of Western liberal democracies as culturally incompatible with traditional conservative values.

## Legitimacy sabotage: Undermining the West's influence

One of Russia's goals is to undermine the cultural, social, and political influence of Western liberal democracies in CEE. To achieve this goal, Russia uses gender-based disinformation to show that the cultural influence of these countries is illegitimate. Put differently, Russia uses gender-based disinformation to demonstrate that the cultural influence of states like France, Germany, and the US on CEE states is improper because it is destructive, manipulative, and at odds with local conservative norms.

To undermine the moral legitimacy of Western liberal democracies, Russian disinformation claims that the West's progressive values cause negative outcomes. The goal of this strategy is to convince targeted states that fostering closer ties with “progressive” Western states is at odds with their local conservative values. Three narratives support this goal. They suggest that citizens in Western liberal democracies confuse gender roles and identities, that progressive norms are ruining society, and that these Western states push their gender ideology onto others.

Analysis of our dataset requires consideration of a few examples of legitimacy sabotage in practice. One article suggested that Western schools teach students that Jesus was bisexual.^50^ Another article discussed how Western women were being taught to act like men and reject their femininity.^51^ Other articles suggested that the West's cultural influence leads men to lose their manliness^52^ and children to lose their mothers and fathers.^53^ These articles use ideas about gender and sexuality to argue that the cultural influence of Western liberal democracies is illegitimate because it conflicts with traditional and conservative family values.

Across these three narratives, two overarching themes are evident. The first portrays Western liberal democracies as decadent, dysfunctional, and rotten. These articles claim that the progressive values championed by some Western liberal democracies are immoral. Several articles falsely claimed that Western European countries were close to legalizing pedophilia, attacking families, and encouraging young children to change their sexes.^54^ One article claimed that Emmanuel Macron is gay and presented him as a symbol of the West's moral decay.^55^ Others described Western societies as having a “craving for lewdness” and decadence.^56^ Some narratives broadened this critique to Western Europe as a whole, claiming that the region was “immoral” because of its rejection of Christian family values.^57^ These narratives were carefully crafted to resonate with conservative Christian audiences. By framing Western liberal democracies as morally degenerate, they create a sharp values-based distinction between Western norms and social conservatism. Russia's goal is to convey that the cultural influence of Western liberal democracies violates social conservative norms and is therefore illegitimate.

A second theme in Russian gender-based disinformation speaks directly to the cost of engaging with progressive Western states. One of the key themes across Russia's disinformation is that interacting with Western liberal democracies, or adopting their cultural values, causes negative social outcomes. For example, some articles claimed that the Coronavirus was caused by gay marriage.^58^ Other articles suggested that agents from Western democracies target countries like Russia, Georgia, and Belarus with policies that are designed to destroy their core cultural values. One article claimed that Western liberal democracies were trying to feminize men in Belarus.^59^ A separate article suggested that Western activists were trying to humiliate Serbian family men.^60^ Yet another claimed that Western liberal democracies brought sodomy to Belarus.^61^ Consistent with Russia's goal of undermining the US’ influence over Europe, several articles highlighted the cultural consequences of engaging with the US specifically. According to these disinformation narratives, American proxies influence elections to promote gay rights, sex reassignments, and gender-progressive politicians.^62^ Others suggested that American social networks teach sodomy, and that American embassies promote sex between minors and force host countries to hold pride parades.^63^ These narratives remain salient in the contemporary context. As American political discourse renews its emphasis on traditional values, Russia frames the cultural backlash in the United States as further confirmation of the social costs of liberal engagement.

By arguing that Western liberal democracies suffer from moral rot, Russia seeks to undermine the legitimacy of rival governments. It provides strong and clear examples of why any country, but particularly countries with conservative populations, should not foster close cultural, social, and political ties with Western liberal democracies. By attacking the West's morality, Russian disinformation makes a strong argument about why non-Western states should not emulate, and even resist, the West's influence.

## Legitimacy sabotage: Undermining NATO and the EU

Russia also uses gender-based disinformation to undermine the moral legitimacy of NATO and the EU. Consistent with Ornstein's claim that Russia seeks to break apart these international organizations from within,^64^ we find evidence that Russia tries to undermine support for these institutions with gender-based disinformation. Russia uses disinformation to attack NATO by making claims that NATO forces cause more harm than good, implying that NATO's use of force is illegitimate. When Russia uses disinformation to attack the EU, it paints it as an illegitimate political authority because it has softened European borders and does not adequately address the consequences. We identified two narratives that speak directly to these objectives: (1) that NATO-aligned armed forces perpetrate sexual violence; and (2) that migrants in the EU commit sexual violence and threaten the civilians of all EU countries.

The narrative that NATO-aligned states perpetrate sexual violence takes elements of the truth and distorts them to suit Russia's needs. The professional armed forces of several prominent NATO member-states are undergoing a culture crisis in which military sexual violence is increasingly understood as a systemic issue.^65^ However, the way Russia discusses military sexual violence does not seek to hold these armed forces accountable for their actions. Rather, Russia fabricates claims to advance its foreign policy agenda.

The narratives about NATO-aligned forces perpetrating sexual violence suggest that NATO does more harm than good and should not be trusted. One article suggests that Western mercenaries in Ukraine will rape and kill civilians.^66^ Another article tells a story about a human trafficker who works to provide young girls for NATO forces to rape when they are deployed.^67^ A third article suggests that American soldiers deployed at a military base in Macedonia will be able to rape locals without any legal consequences.^68^ These pernicious narratives are designed to evoke fear and reduce public support for NATO-aligned forces and NATO's missions and operations. By spreading disinformation about the crimes NATO-aligned armed forces perpetrate against locals, Russia seeks to undercut public support for NATO forces on their country's territory.

Russia also seeks to foster Euroscepticism. Articles designed to achieve this objective were published primarily between 2017 and 2018, when European countries such as Germany were in the process of settling predominantly Syrian refugees. Russia used the refugee crisis and the fear that the migrants who took refuge in Germany would spread through the Schengen area to foster Euroscepticism. The dataset contains several articles that suggest that the influx of migrants will lead to higher rates of sexual violence throughout Europe. For instance, one article suggests that refugees in Germany have committed over 500 sex crimes within the past two years.^69^ Sweden, another country that took in a substantial number of refugees, is described by another article as experiencing a 1000% increase in incidents of reported rapes because of the migrants it hosted between 2015 and 2017. In reality, the number of reported rapes increased by 1.4%.^70^ Articles that suggest migrants perpetrate sexual violence dovetail with another set of articles that suggest European authorities are unwilling to investigate sexual assault charges when the alleged perpetrator is a migrant. These articles suggest that European institutions allow migrant-rapists to travel throughout Europe, nobody is safe, and police officers and courts of law are unwilling to investigate. By connecting fabricated stories of sexual violence to xenophobic views of immigrants, Russia is trying to undermine support for the EU among the citizens of its member states.

Anti-NATO and EU sentiment is not just limited to these two narratives about sexual violence. Across the other six narratives, we find evidence that Russia uses moral legitimacy sabotage to prevent NATO and the EU from expanding. A common message across narratives is that if a country wants to join NATO or the EU, it will need to sacrifice its conservative values. Several narratives suggested that if Georgia, Azerbaijan, and Armenia want to join NATO and the EU, they will need to legalize gay marriage and hold pride parades.^71^ These articles often use fabricated or misleading historical evidence from existing member-states to illustrate to non-members what will happen if they join. One article claimed that Europeans are forced to be gay.^72^ Another article claimed that the EU forced Italy to legalize gay marriage and punished Italy for supporting traditional values.^73^

Taken together, these narratives illustrate how Russia strategically uses gender-based disinformation as a tool to undermine the moral legitimacy of NATO and the EU. By portraying NATO forces as perpetrators of sexual violence and by framing EU migration policy as enabling sexual violence by migrants, Russia seeks to erode public trust in these institutions’ capacity to provide security and uphold shared values. Moreover, by weaponizing narratives about gender and sexuality – such as suggesting that EU or NATO membership requires adopting liberal social policies that allegedly threaten conservative values – Russia aims to deepen societal divisions and foster resistance to Euro-Atlantic integration. In this way, gender-based disinformation functions not merely as sensational propaganda but as a deliberate strategy to delegitimize NATO and the EU from within, weaken their cohesion, and obstruct their efforts to expand or maintain influence. Ultimately, these disinformation campaigns serve Russia's broader geopolitical goal of fragmenting Western alliances.

## Legitimizing Russia's foreign policy goals

Russia also uses gender-based disinformation to legitimize its foreign policy goals. We found three narratives that Russia uses to justify its use of force. Russia claims that it wages war in a humane way, that any information about Russian human rights abuses is fabricated by the West, and that Russia must defend against progressive ideals and eroding family values in other countries. These articles were predominantly in Russian, but were also found in languages including English, Italian, Hungarian, French, and Arabic, suggesting that Russian legitimation strategies are aimed at both internal and external audiences. Central to the first two narratives are claims about sexual violence. Russia consistently makes the false claim that its soldiers do not perpetrate sexual violence, and that when there are reports of sexual violence, they are fabricated by Western liberal democracies. The implicit message in these kinds of articles is that the West is fabricating stories that portray women and children as victims so that it can intervene to prevent Russia from achieving its foreign policy goals.

Russia also employs ad hominem attacks to criticize the character of those who hold Russia accountable. These attacks are commonly used when reports of sexual violence highlight the actions of a particular individual, rather than something systemic. For example, one article suggested that a whistleblower who released video evidence of Russian soldiers committing sexual violence should not be trusted. The article claimed that he was not a true whistleblower because a newspaper had paid him for the video.^74^ The article's overarching claim was that whistleblowers speak truth to power because it is the right thing to do, not for financial gain. Because the evidence against Russian soldiers was indisputable, Russia began making personal attacks against the whistleblower that were unrelated to the integrity of the video to suggest that civilians should disregard his video evidence. Other articles claimed that the International Criminal Court's investigation into Putin was illegitimate because it was led by a pedophile.^75^ In both sets of articles, the disinformation narrative attacked the character of a person instead of the broader argument the article was defending against.

A second key message in the legitimating articles is that Russia is a force for good in the world that must intervene to save its neighbours from the West's perverse cultural influence. Tapping into narratives about Western values undermining society, several articles claimed that Russia is the only civilization capable of saving the world from collapse. In fact, Russia makes appeals to moral legitimacy to justify its invasion of Ukraine. Specifically, Russia claims that it is fighting to defend traditional values in Ukraine for the benefit of the world.^76^ Russia maintains that Ukraine is rewriting gay rights into its war history, that the Ukrainian population will decline because of homosexuality, and that Ukraine is becoming a gay colony of the West.^77^ Another article claimed that Russia needed to save Ukraine from the American biolaboratories that were developing substances that would make all sex and gender differences disappear.^78^ By spreading false information about the salience of progressive values in Ukraine, Russia can justify its illegal invasion to social conservatives by claiming its actions are designed to save Ukraine from the West's perverse cultural influence.

This section demonstrates how Russia strategically deploys gender-based disinformation as a hybrid tactic to sabotage the legitimacy of Western liberal democracies and to legitimize its own foreign policy objectives. By portraying progressive gender norms as corrosive and foreign institutions as spreaders of moral decay, Russia crafts narratives that cast Western states as decadent, dangerous, and unworthy of emulation. It also presents itself as a moral bulwark—a defender of traditional values and societal stability. These efforts are not limited to domestic audiences. Russia disseminates these narratives globally, adapting them to local contexts to undermine the legitimacy of its adversaries and strengthen perceptions about the legitimacy of its own foreign policy actions.

## Targets of Russian gender-based disinformation

Thus far, we have shown that Russia uses gender-based disinformation to undermine the legitimacy of Western liberal democracies, make organizations like NATO and the EU appear illegitimate, and justify its own military aggression. These campaigns exploit culturally sensitive flashpoints such as policy debates over LGBTQ rights, race, and gender equality, to polarize societies and undermine trust in public institutions.^79^ This is also done more surgically, targeting the contributions of allies to NATO operations. For example, during the early days of Canada's leading the NATO battlegroup in Latvia, Russian sources circulated false claims regarding Canadian military personnel and conflict-related sexual violence to delegitimize Canada's role in Enhanced Forward Presence.^80^ In the domestic political context, these tactics are also ubiquitous, seeking to exacerbate polarization over gender issues via online communities and forums.^81^ In this section, we delve more deeply into a subset of Russian gender-based disinformation where countries with large conservative populations are targeted. By targeting countries with large socially conservative populations, Russia connects pre-existing beliefs about morality with its foreign policy to legitimate itself and delegitimate its adversaries. We thus propose an additional theoretical mechanism to help us understand increasingly sophisticated and discerning disinformation tactics.

As Schmitt highlights, effective disinformation must tap into pre-existing narratives, myths, and identities.^82^ We find some evidence that Russia targets countries with large conservative segments. Across the coded articles, we found that gender-based disinformation was disseminated primarily in Russian, Georgian, Armenian, and Italian. Table 1 summarizes all the countries in which at least fifteen percent of the population speaks these languages.

**Table 1.** Primary targets of pro-kremlin gender-based disinformation^a^

Country	Target language population (%)	Accept homosexuality (%)	Consider divorce acceptable (%)
Belarus	92	10	43
Russia	81	9	45
Latvia	29	17	40
Estonia	26	20	44
Kazakhstan	21	6	28
Ukraine	15	6	38
Italy	96	47	54
Georgia	82	2	10
Armenia	98	2	13

a To compile the table, we identified only the four most common languages that Russian disinformation was in. The countries in the table are the countries in the world that have at least 15% of their population that speak the language. Estimates from EPR. See: Manuel Vogt, Nils-Christian Bormann, Seraina Ruegger, Lars-Erik Cederman, Philipp Hunziker, and Luc Girardin, “Integrating Data on Ethnicity, Geography, and Conflict: The Ethnic Power Relations Data Set Family,” *Journal of Conflict Resolution* 59, no. 7 (2015): 1327-42. Values are for Russian and Russian speakers and the titular ethnic groups in Italy, Georgia, and Armenia. Acceptance of homosexuality and divorce acceptability are calculated according to what the World Value Survey calls “high”. See: Christian Haerpfer, Ronald Inglehart, Alejandro Moreno, Christian Welzel, Kseniya Kizilova, Jaime Diez-Medrano, Marta Lagos, Pippa Norris, Eduard Ponarin, and Bjorn Puranen, “World Values Survey: Round Seven - Country-Pooled Datafile Version 6.0.,” (Madrid, Spain and Vienna, Austria: JD Systems Institute and WVSA Secretariat, 2022).

Except for Italy, all nine countries are in Eastern Europe and have populations that espouse largely conservative values. Consider the proportion of people who accept homosexuality. In six of the nine countries, support for homosexuality is 10 percent of the population or less. Across all nine countries, an average of 13 percent of people accept homosexuality. Even in Italy, the clear outlier, less than half the population thinks that homosexuality is socially acceptable. By comparison, 68 and 69 percent of the population in Great Britain and Canada, respectively, accept homosexuality. These figures highlight that an overwhelming majority of people in these nine countries favour traditional values.

The acceptability of divorce can be considered as a second metric. Although it is an imperfect proxy of family values, people who think divorce is acceptable may be more likely to favour an individualist approach to marriage, one that prioritizes personal happiness over traditional family duty. In other words, countries where divorce is more socially acceptable are likely to be less susceptible to disinformation that capitalizes on traditional family values. Across all nine countries, only 35 percent of people think that divorce is acceptable. Again, Italy appears to be an outlier, but in Georgia and Armenia, roughly one in ten people think divorce is socially acceptable. This data is further evidence that some of the countries targeted with Russian gender-based disinformation have large conservative segments that tend to favour traditional family values.

The fact that Russia is targeting countries with high support for traditional values is preliminary evidence that Russia is not just trying to polarize society or undermine democratic resilience, as Obradovic and Neathery-Castro suggest.^83^ If Russia's only goal was to polarize its adversaries, we would expect that Russia would *only* target countries where support for traditional values is a significant wedge that roughly divides the population in half. Russia is certainly doing that by aiming disinformation campaigns against liberal democracies, but the table above highlights that Russia also targets countries where the population already holds largely conservative values. Except for Italy, all eight countries in Eastern Europe have a common connection to the Orthodox Christian Church, which tends to espouse the same traditional values as Russia.

By targeting countries with large conservative populations, Russia's goal is distinct from its attempt to polarize societies in liberal democracies. Instead, Russia legitimates its worldview by spreading narratives that illustrate that they champion the values espoused by conservatives. At the same time, Russia taps into conservative values to sabotage the legitimacy of its adversaries by highlighting that they are at odds with conservative values. Russian disinformation suggests that if Georgia and Armenia want to join NATO or the EU, they will need to legalize gay marriage and hold pride parades. Only two percent of people in these countries think homosexuality is acceptable.^84^ As this example illustrates, Russian disinformation taps into conservative values to undermine its foreign adversaries and promote its geopolitical goals. By contrast, when Russian disinformation targets liberal democracies, disinformation serves different, often context-specific, goals. For example, in countries like Canada, worsening partisanship is one observable objective of disinformation.

While we acknowledge the geopolitical insecurities on the Eastern flank and that Russia targets liberal democracies all over the world, we maintain that there is preliminary support for an additional theoretical mechanism in the disinformation debate: Russia strategically targets countries with large, socially conservative populations to amplify and weaponize pre-existing traditional values.^85^ By aligning itself with conservative moral worldviews, Russia legitimates its own regime and foreign policy but also delegitimizes Western states and institutions, portraying them as morally decadent and incompatible with local values. This is a distinct logic compared to what has previously been identified in the literature, which focused primarily on how Russian disinformation aims to sow division or polarize evenly split societies. To be sure, even when gender-based disinformation operates as a powerful tool for forging ideological alignment and strengthening Russia's soft power, it is still doing so to curtail Western geopolitical ambitions. These patterns underscore the importance of considering local value systems when evaluating the effectiveness and dissemination of foreign disinformation campaigns.

## Conclusion

We have shown that Russia uses gender-based disinformation as a deliberate and strategic component of its hybrid warfare toolkit. Specifically, Russia uses a set of common narratives to undermine the legitimacy of Western states and institutions and strengthen public perceptions of itself. These findings reveal that gender-based disinformation is not merely a byproduct of Russian propaganda but a deliberate hybrid tool. By exploiting cultural anxieties and weaponizing narratives around gender, Russia seeks to increase support for its foreign policy goals, strengthen its sphere of influence, and undermine the legitimacy of its adversaries.

As global debates over gender identity and rights intensify, these disinformation campaigns are likely to become more sophisticated and more difficult to counter. It is evident that gender-based disinformation is not simply a tool of domestic political suppression. It is also a strategic instrument of statecraft, deployed to weaken adversaries’ legitimacy, sow discord, and project alternative value systems. It functions in part by way of weaponizing gendered narratives, such that states have subtly challenged liberal norms, called into question the moral legitimacy of strategic competitors, and advanced foreign policy agendas under the guise of cultural preservation or moral superiority. This raises critical questions for policymakers and scholars alike: How can liberal democracies defend against narratives that exploit deeply rooted cultural values without reinforcing the very divisions they seek to mend? What mechanisms can international institutions develop to anticipate and counter gendered disinformation before it corrodes trust and legitimacy?

Future research should explore comparative cases, examine the interplay between gendered narratives and other ideological tropes, and assess the long-term implications for international security and democratic resilience. This article identifies the strategic logic of gender-based disinformation narratives. Future research could apply methods of statistical inference to identify patterns in both space and time. In short, gender-based disinformation is a key pillar of contemporary hybrid warfare, and its strategic significance will likely grow in the years ahead.

## Notes

1. Alexander Lanoszka, "Disinformation in International Politics," *European Journal of International Security* 4, no. 2 (2019): 227–48.

2. Frank Gardner and Paulina Kola, "Russia could attack Nato within five years, says alliance chief in stark new warning," *BBC News,* 11 December 2025, https://www.bbc.com/news/articles/cn81×8py3j5o.

3. Mark Rutte, "Keynote speech by NATO Secretary General Mark Rutte and moderated discussion with Minister for Foreign Affairs of Germany Johann Wadephul," *NATO*, 11 December 2025, https://www.nato.int/en/news-and-events/events/transcripts/2025/12/11/keynote-speech-by-nato-secretary-general-mark-rutte-and-moderated-discussion-with-the-minister-for-foreign-affairs-of-germany-johann-wadephul.

4. Meghan Davidson Ladly, "How Iran Targets Women Dissidents Abroad," *New Lines Magazine*, 21 February 2025, https://newlinesmag.com/spotlight/how-iran-targets-women-dissidents-abroad/.

5. Lana Obradovic and Jody Neathery-Castro, *Integrating Gender Perspective in Cognitive Warfare*, Allied Command Transformation/Strategic Plans and Policy (Brussels, 2023), https://issuu.com/sppplp/docs/openpublicationintegratinggenderperspectivein.

6. Andrew M. Guess and Benjamin A. Lyons, “Misinformation, Disinformation, and Online Propaganda,” in *Social Media and Democracy: The State of the Field, Prospects for Reform*, ed. Nathaniel Persily and Joshua A. Tucker (Cambridge University Press, 2020), 11.

7. Ibid.

8. Dhanaraj Thakur and DeVan Hankerson Madrigal, *Facts and their Discontents: A Research Agenda for Online Disinformation, Race, and Gender* (Center for Democracy & Technology, 2021).

9. Barbara Mithani and Jasmine Rodriguez, "AI enters Congress: Sexually explicit deepfakes target women lawmakers," *The 19th,* 11 December 2024, https://19thnews.org/2024/12/ai-sexually-explicit-deepfakes-target-women-congress/.

10. Elizabeth Goodyear-Grant, *Gendered News: Media Coverage and Electoral Politics in Canada* (UBC Press, 2013); Linda Trimble, Jennifer Curtin, Angelia Wagner, Meagan Auer, VKG Woodman, and Bethan Owens, “Gender novelty and personalized news coverage in Australia and Canada,” *International Political Science Review* 42, no. 2 (2021): 164–78.

11. Irene Kahn, *Report of the Special Rapporteur on the promotion and protection of the right to freedom of opinion and expression*, UN General Assembly (7 August 2023).

12. Mona Lena Krook, *Violence Against Women in Politics* (Oxford University Press, 2020); Philip R. Kavanaugh, “The Continuum of Sexual Violence: Women's Accounts of Victimization in Urban Nightlife,” *Feminist Criminology* 8, no. 1 (2013): 20–39; Gabrielle Bardall, Elin Bjarnegård, and Jennifer M. Piscopo, “How is Political Violence Gendered? Disentangling Motives, Forms, and Impacts,” *Political Studies* 68, no. 4 (2020): 916–35.

13. Sjoberg, Laura, “Gender, structure, and war: what Waltz couldn't see,” *International Theory 4* no. 1 (2012): 20–21.

14. Woojeong Jang, “Women against Putin: gendered security threats and female leaders,” *European Journal of International Relations* 31, no. 3 (2025): 681–714.

15. Samantha Bradshaw and Amélie Henle, “The Gender Dimensions of Foreign Influence Operations,” *International Journal of Communication* 15, no. 1 (2021): 4596–4618.

16. Lana Obradovic and Jody Neathery-Castro, *Integrating Gender Perspective in Cognitive Warfare* (2023), https://issuu.com/sppplp/docs/openpublicationintegratinggenderperspectivein.

17. Bradshaw and Henle, “The Gender Dimensions of Foreign Influence Operations.”

18. Ibid.

19. Ellen Judson, Asli Atay, Alex Krasodomski-Jones, Rose Lasko-Skinner, and Josh Smith, “Engendering Hate: The Contours of State-Aligned Gendered Disinformation Online,” DEMOS (London, 2020).

20. Frank Gardner, “MI6 chief: 'We are operating in space between peace and war',” *BBC News* 2025, https://www.bbc.com/news/articles/cgqzed1vjw5o.

21. Ibid.

22. NATO, “NATO Policy on Women, Peace and Security,” 2024 (accessed 3 December 2025), https://www.nato.int/natostaticfl2014/assets/pdf/2024/7/pdf/240711-WPS-Policy-2024en.pdf.

23. UN Security Council, “9760th Meeting,” *The United Nations* (2024), https://digitallibrary.un.org/record/4065302?ln=en&v=pdf.

24. Bradshaw and Henle, “The Gender Dimensions of Foreign Influence Operations.”

25. Lucina Di Meco and Kristina Wilfore, “Gendered disinformation is a national security problem,” *Brookings,* 8 March 2021, https://www.brookings.edu/articles/gendered-disinformation-is-a-national-security-problem/.

26. Global Affairs Canada, "Canada's response to the Islamic Republic of Iran," *Government of Canada,* 2019, https://www.international.gc.ca/country-pays/iran/relations.aspx?lang=eng.

27. The EU imposes gender neutrality on Georgian kindergartens,” EUvsDisinfo, 27 May 2019 (accessed 3 June 2025), https://euvsdisinfo.eu/report/eu-imposes-gender-neutrality-on-georgian-kindergartens/.

28. Alice Eagly, “Gender Roles,” in *Encyclopedia of Psychology and Religion*, ed. Alan E. Kazdin (Oxford University Press, 2000), 448–53.

29. Deborah Rudacille, *The Riddle of Gender: Science, Activism, and Transgender Rights* (Anchor Books, 2006).

30. Jay J. Van Bavel, Steve Rathje, Madalina Vlasceanu, and Clara Pretus, "Updating the identity-based model of belief: From false belief to the spread of misinformation," *Current Opinion in Psychology* 56 (2024): 101787.

31. Brittany S. Liu and Peter H. Ditto, "What Dilemma? Moral Evaluation Shapes Factual Belief," *Social Psychological and Personality Science* 4, no. 3 (2013): 316–23.

32. Ibid.

33. Marc C. Suchman, “The Academy of Management Review,” *The Academy of Management Review* 20, no. 3 (1995): 574.

34. Suzanne Nossel, “Going Legit,” *Democracy: A Journal of Ideas*, no. 3 (2007).

35. Robert Kagan, “America's Crisis of Legitimacy,” *Foreign Affairs* 83, no. 2 (2004): 76.

36. Christian Reus-Smit, “International Crises of Legitimacy,” *International Politics* 44, no. 1 (2007): 158; Ian Clark, *Legitimacy in International Society* (Oxford University Press, 2007), 4.

37. Erik Voeten, "The Political Origins of the UN Security Council's Ability to Legitimize the Use of Force," *International Organization* 59, no. 3 (2005): 527–57; Reus-Smit, “International Crises of Legitimacy.”

38. T.V. Paul, *Restraining Great Powers: Soft Balancing from Empires to the Global Era* (Yale University Press, 2018).

39. “Database,” 2026 (accessed 23 March 2026), https://euvsdisinfo.eu/.

40. See narrative identification methodology in Megan MacKenzie, *Good Soldiers Don't Rape: The Stories We Tell about Military Sexual Violence* (Cambridge University Press, 2023).

41. Mitchell A. Orenstein, *The Lands in Between: Russia vs. the West and the New Politics of Hybrid War* (Oxford University Press, 2019), 34.

42. V. Gerasimov, “The Value of Science Is in the Foresight: New Challenges Demand Rethinking the Forms and Methods of Carrying out Combat Operations,” *Military Review* (2016).

43. Margot Light, “Russia and the EU: Strategic partners or strategic rivals,” *Journal of Common Market Studies* 46, no. 1 (2008): 7–27.

44. Orenstein, *The Lands in Between*.

45. Ibid.

46. Laura Rosenberger and Thomas Morley, “Russia's Promotion of Illiberal Populism: Tools, Tactics, Networks,” *Alliance for Securing Democracy* (2019).

47. William C. Ashmore, “Impact of alleged Russian cyber attacks,” *Baltic Security & Defence Review* 11, no. 1 (2009): 4–40.

48. Andrew Wilson, *Ukraine's Orange Revolution* (Yale University Press, 2005).

49. Orenstein, *The Lands in Between*.

50. Article 107.

51. Article 134.

52. Articles 159; 333.

53. Articles 10; 294.

54. Articles 268; 288; 365.

55. Article 346.

56. Article 25; 144; 145; 147.

57. Articles 87; 123.

58. Articles 138; 346.

59. Article 265.

60. Article 8.

61. Article 58.

62. Articles 45; 51; 81; 89; 142; 289.

63. Articles 142; 27.

64. Orenstein, *The Lands in Between*.

65. MacKenzie, *Good Soldiers Don't Rape: The Stories We Tell about Military Sexual Violence*.

66. Article 197.

67. Article 232.

68. Article 231.

69. Article 238.

70. Article 245.

71. Articles 80; 36; 67; 28; 42; 266.

72. Article 77.

73. Articles 82; 360.

74. Article 214.

75. Articles 259; 2; 99.

76. Article 86.

77. Articles 29; 33; 170; 96; 72.

78. Article 289.

79. Owen Wong, Claire Mountford, Megan Barker, and Stéfanie von Hlatky, *How NATO Should Respond to Russian Gender-Based Disinformation* (Queen's University Centre for International and Defence Policy, October 2025), https://www.queensu.ca/cidp/publications/policy-briefs/how-nato-should-respond-russian-gender-based-disinformation.

80. Owen Wong, *Gender-Based Disinformation: A Tool of Hybrid Warfare* (Queen's University Centre for International and Defence Policy, February 2024), https://www.queensu.ca/cidp/publications/policy-briefs/gender-based-disinformation-tool-hybrid-warfare.

81. Stéfanie von Hlatky, *Deploying Feminism: The Role of Gender in NATO Military Operations* (Oxford University Press, 2022; 2025).

82. Olivier Schmitt, “When are strategic narratives effective? The shaping of political discourse through the interaction between political myths and strategic narratives,” *Contemporary Security Policy* 39, no. 4 (2018): 487–511.

83. Obradovic and Neathery-Castro, *Integrating Gender Perspective in Cognitive Warfare*.

84. “LGBT Rights in Armenia,” Equaldex, 2025, https://www.equaldex.com/region/armenia.

85. Given the scope of the dataset and EU vs. Disinformation's sampling strategy, we cannot make claims about whether countries with more conservative populations are targeted by gender-based disinformation. Our aim is more limited: to qualitatively show that Russia appears to target countries with large conservative populations.

